# Tumor Suppressor *LINC02487* Inhibits Oral Squamous Cell Carcinoma Cell Migration and Invasion Through the USP17–SNAI1 Axis

**DOI:** 10.3389/fonc.2020.559808

**Published:** 2020-10-29

**Authors:** Lu Feng, Jianjun Zhang, Minglei Sun, Feng Qiu, Wantao Chen, Weiliu Qiu

**Affiliations:** ^1^ Department of Oral and Maxillofacial Surgery, The First Affiliated Hospital of Zhengzhou University, Zhengzhou, China; ^2^ Department of Oral and Maxillofacial-Head Neck Oncology, Ninth People’s Hospital, Shanghai Jiao Tong University School of Medicine, Shanghai Key Laboratory of Stomatology, Shanghai, China

**Keywords:** long noncoding RNA, oral squamous cell carcinoma, tumor suppressor, deubiquitinating enzyme, migration, invasion

## Abstract

**Purpose:**

The aim of this study was to explore the functions and associated mechanisms of long noncoding RNA *LINC02487* in oral squamous cell carcinoma (OSCC).

**Methods:**

The relative expression levels of *LINC02487* in OSCC cell lines and tissue samples were examined by RT-qPCR. Intracellular localization was determined using RNA fluorescence *in situ* hybridization. *LINC02487* was cloned into the pCMV-puro vector and then introduced into OSCC cells using lentiviral transfection. Cell processes, such as proliferation, apoptosis, migration, and invasion, were subsequently examined. *LINC02487*-binding proteins were identified by ChIRP–MS and confirmed by RNA immunoprecipitation. Protein expression was determined by western blotting assay.

**Results:**

*LINC02487* has been reported to be downregulated in OSCC. Here, we confirmed that the expression of *LINC02487* was reduced in 6 OSCC cell lines compared with that in immortalized normal oral epithelial cells and in 50 OSCC samples compared with paired adjacent normal tissue in a Chinese population and that *LINC02487* expression levels were associated with cancer metastasis. We further identified that *LINC02487* was localized to the cytoplasm, aggregated around the nuclear membrane. Functional studies demonstrate that overexpression of *LINC02487* significantly suppresses cell migration and invasion and also inhibits cell proliferation. For the mechanism, we reveal that *LINC02487* directly binds to USP17, a deubiquitinating enzyme, and regulates cell migration and invasion through the USP17–SNAI1 axis in a process that involves epithelial–mesenchymal transition (EMT).

**Conclusion:**

Our results confirm that long noncoding RNA *LINC02487* is downregulated in OSCC tissue samples and cell lines. We also find that *LINC02487* acts as a tumor suppressor through the USP17–SNAI1 axis.

## Introduction

Oral squamous cell carcinoma (OSCC) is the most frequently diagnosed cancer of the oral cavity and oropharynx and ranks as the sixth most common malignancy in Asia. Nearly 274,300 new cases are diagnosed each year, accounting for approximately 3.8% of all cancer cases (1–3). According to recent data, China has an estimated 48,100 new OSCC cases and 22,100 OSCC-related deaths per year (4, 5). Tobacco smoking and alcohol consumption are the main risk factors for the disease, and together are responsible for approximately 75% of OSCCs. Betel quid chewing and viral infections, such as that with high-risk human papillomavirus (HPV), are also considered causative of OSCC (6). Few improvements have been made to the overall prognosis for advanced-stage OSCC in the past two to three decades, resulting in a heavy disease burden for patients and their families (7, 8). Identifying efficient biomarkers and therapeutic targets as well as better understanding the mechanisms underlying OSCC development and progression continue to be the focus of intensive research.

It is estimated that more than 70% of the human genome can be transcribed; however, most transcripts do not serve as blueprints for protein synthesis (9). Analysis of the most recent GENCODE release indicates that only 34% of the human genome comprises protein-coding genes, and the rest consists of noncoding genes, including loci for small noncoding RNAs (sncRNAs, <200 nt), long noncoding RNAs (lncRNAs, >200 nt), and pseudogenes (10). The human genome contains more than 15,000 lncRNA genes that give rise to more than 28,000 lncRNA transcripts, comprising nearly 14% of the total number of transcripts (GENCODE) (10). Small noncoding RNAs, such as microRNAs, are well characterized and have been shown to have important roles in cancer, including oral cancer (11–14). Meanwhile, lncRNAs have gained increasing interest in the last two decades and have potential as diagnostic and/or prognostic biomarkers for cancer, including OSCC (15–17).

Long noncoding RNA *LINC02487*, previously known as *LOC441178*, is 2557 bp long and located in chromosome 6. This lncRNA is reportedly downregulated in OSCCs in a study that used a microarray data set consisting of 167 cancers and 45 healthy oral mucosae, which was further validated in three independent data sets (18). Its low expression level in OSCC was also found to be associated with shorter postoperative survival time (19). Given that the differences in expression were stable and significant, *LINC02487* has the potential for use as a biomarker in oral cancer diagnosis and prognosis. However, the precise role and underlying functional mechanism of *LINC02487* in OSCC development remain unknown. In this study, we confirm that the expression of *LINC02487* was dysregulated in OSCC samples from patients from a Chinese population and explore the biological functions of *LINC02487* in OSCC as well as the underlying molecular mechanisms, using several oral cancer cell lines.

Patients with advanced OSCC often progress to have local cervical lymph node involvement or distant metastasis, which are responsible for most cancer-related deaths (8, 20). Epithelial–mesenchymal transition (EMT) plays a critical role during cancer progression and is positively correlated with poor prognosis in cancer patients. The main features of EMT are the loss of epithelial characteristics (loss of cell–cell contact, cytoskeletal remodeling, polarity changes) and the acquisition of a mesenchymal phenotype, which result in increased cell motility and invasion, enhanced migratory capacity, and elevated resistance to apoptosis, eventually leading to distant metastasis (21–23). Recent evidence indicates that lncRNAs may be involved in EMT and tumor invasion-metastasis cascade *via* epigenetic modifications (24, 25). For example, lncRNA *SATB2-AS1* suppresses cancer progression through the regulation of SNAI1 expression in colorectal cancer (26), and lncRNA *ELIT-1* promotes EMT through the TGFβ/SMAD signaling pathway by acting as a SMAD3 cofactor (27). In this study, we also explore the connection between *LINC02487* and EMT in OSCC metastasis.

## Materials and Methods

### Patient Samples and Public Databases

This study was approved by the Ethics Committee of Zhengzhou University. Tissue samples (including cancerous and paired adjacent normal tissues) and the corresponding clinical/pathological data were obtained from OSCC patients of the Sharing Platform for the Tissue Sample and Bioinformatics Database of Oral Maxillofacial Tumor (http://mdl.shsmu.edu.cn/OMNDB/page/home/home_en.jsp; Shanghai, China). This platform contains samples from the Department of Oral and Maxillofacial-Head and Neck Oncology, Ninth People’s Hospital, Shanghai Jiao Tong University School of Medicine, China. Written informed consent was obtained from all the participants. Fifty pairs of samples were included in this study, and their clinical characteristics are summarized in **Table 1**. Relative *LINC02487* expression data for head and neck squamous cell carcinoma (HNSCC) patients and healthy controls were obtained from TANRIC, an open-access resource for interactive exploration of lncRNAs in cancer (https://ibl.mdanderson.org/tanric/_design/basic/main.html; data collected in 2018.08.06) (28), following the website’s instructions and using data sets from The Cancer Genome Atlas (TCGA) (29).

**Table 1:** The relationship between relative expression of *LINC02487* and clinical characteristics.

Characteristics		Relative Expression	*P*
Gender	male(n=32)	0.079 ± 0.020	0.068
	female(n=18)	0.158 ± 0.043	
Age	≥60ys(n=34)	0.112 ± 0.027	0.365
	<60ys(n=16)	0.097 ± 0.030	
Local invasion	Yes(n=15)	0.081 ± 0.037	0.21
	No(n=35)	0.118 ± 0.025	
Metastasis	Yes(n=26)	0.157 ± 0.040	0.03^*^
	No(n=24)	0.070 ± 0.018	
Tumor size	≥4cm(n=13)	0.099 ± 0.044	0.761
	<4cm(n=35)	0.114 ± 0.025	

*P < 0.05

### Cell Lines and Cell Culture

The HEK293T cell line was obtained from the American Type Culture Collection (ATCC; Gaithersburg, MD, USA) and cultured in Dulbecco’s modified Eagle’s medium (DMEM) (Gibco/Invitrogen, Grand Island, NY, USA) supplemented with 10% (v/v) fetal bovine serum (FBS) (Gibco). The OSCC cell lines HN6 and HN30 were obtained from Shanghai Key Laboratory of Stomatology (Shanghai, China) and cultured in DMEM supplemented with 10% FBS, penicillin (100 units/mL), and streptomycin (100 μg/mL) in a humidified incubator with 5% CO_2_. The immortalized normal oral epithelial cell line hTERT-OME was purchased from abm™ (Vancouver, BC, Canada) and cultured in a 1:1 mixture of DMEM and Ham’s F12 medium (Gibco/Invitrogen) supplemented with 10% FBS, penicillin (100 units/mL), and streptomycin (100 μg/mL). The remaining OSCC cell lines (HN4, SCC9, SCC25, and Cal27) were also obtained from the Shanghai Key Laboratory of Stomatology and maintained as previously described (30).

### Quantitative RT-PCR

Total cellular RNA was isolated using RNAiso Plus reagent (Takara Biomedical Technology, Beijing, China), and cDNA was generated using the PrimeScript™ RT reagent Kit (Takara). Real-time qPCR was performed on the Applied Biosystems StepOnePlus Real-Time PCR System (Thermo Fisher Scientific, Waltham, MA, USA) using the TB Green™ Premix Ex Taq™ PCR kit (Takara) following the manufacturer’s instructions. Each sample was assayed in triplicate in 10-μL reaction volumes, and all expression levels were normalized against that of *GAPDH* mRNA unless otherwise specified. The mean threshold cycle (Ct) values were calculated from the triplicate Ct values. Samples that had Ct values with SD >0.35 in their triplicate run were repeated. The sequences of the primers used were as follows: *LINC02487*, Forward GGGTATTTTGTGCTCCCCCA and Reverse CAGGCACTGAAGGTTCGGAT; *USP17*, Forward CTATCATTGCGGTCTTTGTCTCC and Reverse AAGTGATGCTACAGGCAGTGA; *SNAI1*, Forward GCTGCAGGACTCTAATCCAGA and Reverse ATCTCCGGAGGTGGGATG.

### RNA Fluorescence In Situ Hybridization (FISH)

The lncRNA *LINC02487* probe mix was designed and synthesized by RiboBio (Guangzhou, China). Hybridization was performed using the Ribo™ FISH Kit (RiboBio) following the manufacturer’s instructions and then observed using confocal laser microscopy (Nikon, Tokyo, Japan). The 18S ribosomal RNA and *U6* probe mixes were used for quality control.

### Lentivirus-Mediated Overexpression

The pCMV-MCS-PGK-Puro vector (PHY-008, HanYin Biotech, Shanghai, China) was used for the stable overexpression of *LINC02487*. This vector contains a puromycin resistance gene and is introduced into cells using lentiviral transfection. Full-length *LINC02487* was synthesized, amplified, and cloned into the pCMV-puro lentiviral vector (HanYin Biotech). pCMV-puro empty-vector transduction particles were used as controls. Cells were seeded in a 6-well plate at a density of 6×10^5^ cells/well 24 h before infection. The medium was removed, and 10–20 multiplicity of infection (MOI) of MISSION pCMV-LINC02487-puro lentiviral particles and 2 mL of Opti-MEM medium (Gibco) supplemented with polybrene (8 μg/mL, Sigma, St. Louis, MO, USA) was added to the cells. Lentiviral infection was performed at 37°C for 6 h unless intensive cell toxicity was observed. The medium was then replaced with 2 mL of fresh medium. Infected cells were allowed to grow for 48–72 h and then selected with puromycin-containing medium (Sigma). The expression of *LINC02487* was confirmed by RT-qPCR.

### SiRNA or Plasmid Transfection

For the knockdown of *LINC02487* and *USP17*, small interfering RNAs (siRNAs) were designed and synthesized by RiboBio. For transient overexpression of tagged *USP17*, the pCMV-HA-puro vector was used, which contains a C-terminal HA epitope tag. Transfection was performed using Lipofectamine 3000 (Thermo Fisher Scientific) following the manufacturer’s instructions. The expression of *LINC02487* was confirmed by RT-qPCR. The expression of mRNA *USP17* was confirmed by RT-qPCR, and the expression of protein USP17 was confirmed by western blotting.

### Cell Proliferation Assays

Cell proliferation was determined by CCK-8 assay and EdU (5-ethynyl-20-deoxyuridine) staining. For the CCK-8 assay, 1000 cells of different groups/well were seeded into 96-well plates. At each time point (24, 48, 72, and 96 h after seeding), 10 μL of CCK-8 reagent (Beyotime Biotech, Shanghai, China), and 100 μL of medium were added to each well followed by incubation at 37°C for 2 h. The optical density of the wells was measured at 450 nm using a microplate reader (SpectraMax i3, Molecular Devices, San Jose, CA, USA). Data were from three separate experiments, each with three replications. For the EdU staining assay, the Cell-Light™ EdU Cell Proliferation Detection Kit (RiboBio) was used. Cells from different groups were seeded into 96-well plates at the appropriate density. After incubation at 37°C and 5% CO_2_ for 48 h, 50 mM EdU reagent was added to the cells, followed by incubation for another 2 h. The cells were then fixed in 4% paraformaldehyde and stained with Apollo Dye solution to label proliferating cells. Nuclei were counterstained with DAPI. The cell proliferation rate was calculated according to the manufacturer’s instructions. Images were obtained using a fluorescence microscope (Zeiss, Jena, Germany).

### Clone Formation Assay

From each group, 500 cells were plated per well of a 6-well culture plate, three wells per group. The cells were incubated at 37°C for 14 days with the growth medium being replaced every third day. After 14 days, the cells were washed twice with phosphate buffer saline (PBS) and fixed in 4% paraformaldehyde and then stained with 0.5% crystal violet. The number of colonies containing ≥50 cells was counted under a microscope. These experiments were performed in triplicate.

### Cell Apoptosis Assays

Analysis of cell apoptosis was performed using both flow cytometry and caspase-3 detection. For flow cytometry, the Annexin V/FITC Apoptosis Detection Kit (BD Pharmingen, Franklin Lakes, NJ, USA) was used as per the manufacturer’s instructions. The stained cells were analyzed on a FACSCalibur flow cytometer using CellQuestPro software (BD Pharmingen) to determine the rate of cell apoptosis. For the caspase-3 assay, caspase-3 activity was measured according to the rate of cleavage of the chromogenic caspase substrate, Ac-DEVD-pNA (acetyl-Asp-Glu-Val-Asp p-nitroanilide), using the Caspase-3 Activity Assay Kit (Beyotime Biotech). Cells from each group were harvested, and approximately 50 pg of total protein was added to a reaction buffer containing Ac-DEVD-pNA (2 mM), followed by incubation for 2 h at 37°C; the absorbance of the yellow pNA cleavaged from its corresponding precursor was measured at 405 nm using a spectrometer (SpectraMax i3, Molecular Devices). The specific caspase activity, normalized for total protein content of the cell lysates, was then expressed as a fold change compared with the baseline.

### Wound Healing Assay

Cells were cultured in 6-well plates until they had reached 70%–90% confluence. A scratch was made on the bottom of each well using a 10-μL tip to form wounds. The cells were then washed twice with PBS and cultured under the previous conditions. The wounds were imaged at different time points, and the distance migrated by cells from five different areas for each wound was measured. For each visual field, two straight lines were drawn in the front of both sides of the gap, and the average distance was calculated as the average of the distance of the two lines at the left, middle, and right points of the view.

### Cell Migration and Invasion Assays

A total of 4×10^4^ cells were suspended in 100 μL of DMEM without FBS and seeded into the top chamber of 24-well plate transwell inserts (BD Pharmingen) with an 8-µm membrane pore size. Medium containing 10% FBS was placed into the lower chamber as a chemoattractant. After incubating for 24 h, the cells that did not migrate through the pores were manually removed with a cotton swab. Cells at the bottom of the membrane were fixed and stained with a crystal violet solution (Beyotime Biotech) and then counted and imaged under a microscope. Cell numbers were calculated in eight random fields for each chamber, and the average value was calculated. Each experiment was conducted in triplicate. The cell count index was also used as the counting of migrated cells. Stained cells from the bottom of the chambers were eluted with 500 μL of 33% acetic acid; the absorbance of the eluant was read under a microplate reader at 570 nm (SpectraMax i3, Molecular Devices), and the optical density was recorded as the cell count index to reflect cell number. Cell invasion assays were performed using Matrigel (diluted 1:5 with medium without FBS; BD Pharmingen)-coated transwell inserts following the same procedure as described above.

### Chromatin Isolation by RNA Purification (ChIRP)

An optimized ChIRP assay to identify lncRNA-associated proteins was performed following previously described protocols (31–33) with modifications. The EZ-Magna ChIRP™ RNA Interactome Kit (Merck Millipore, Darmstadt, Germany) was used on the immortalized normal oral epithelial cell line hTERT-OME. Twenty antisense DNA tiling probes targeting *LINC02487* were designed using the online probe designer at singlemoleculefish.com (http://www.singlemoleculefish.com/designer.html) and synthesized (Sangong Biotech) with Biotin-TEG at the 3′ end. Probes were separated into two pools (even and odd) to help eliminate nonspecific signals. A LacZ probe set was used as a negative control, and a blank control without probes was used to eliminate background interference. Approximately 1×10^8^ cells were used per reaction. Cells of the four groups (even, odd, LacZ, and blank) were harvested and cross-linked with formaldehyde to preserve RNA interaction, following which the cell pellets were lysed and sonicated in a water bath at 4°C to shear the complex. The cell lysates were incubated with the biotinylated DNA probes and subjected to capture by streptavidin magnetic beads. The RNA was then extracted from the ChIRP samples and quantified by RT-qPCR as quality control. The proteins were subsequently precipitated, separated by bis–tris SDS–PAGE (Invitrogen) for mass spectrometry (ChIRP–MS) (Applied Protein Technology, Shanghai, China), and verified by western blotting (ChIRP–WB). The raw data from the mass spectrometry were analyzed and searched using Mascot in the UniProt database (34). Peptides with a false discovery rate (FDR) <1% were chosen for further data processing.

### RNA Immunoprecipitation (RIP)

RIP was performed as previously described (35–37) with the following modifications. First, the RIP assay was conducted in 293T cells transiently expressing HA-tagged USP17 through plasmid transfection. A total of 2×10^7^ cells were used per reaction. Second, immunoprecipitation was performed by incubating cell lysates containing RNA–protein complexes with anti-HA-coated magnetic beads. Third, biotin-coupled magnetic beads were used as control. Briefly, cells were cross-linked and lysed in the presence of an RNase inhibitor and a protease inhibitor cocktail, followed by sonication on ice. After immunoprecipitation, purified beads containing RNA–protein complexes were washed following standard procedures. The protein samples were retrieved and detected by western blotting as quality control. The RNA samples were extracted using the RNeasy MinElute Cleanup Kit (Qiagen, Düsseldorf, Germany) and detected by RT-qPCR. The results were presented as *LINC02487* fold enrichment compared with the control.

### Antibodies and Western Blot Analysis

Cells from different groups were harvested, rinsed twice with PBS buffer, and then lysed with lysis buffer (150 mM NaCl, 50 mM Tris-HCl pH 7.4, 1 mM EDTA, 1 mM MgCl_2_, 0.5% NP-40, 1 mM Na3VO4, 1 mM NaF, protease inhibitor cocktail). Protein samples were heated to 95°C for 10 min and then resolved by SDS–PAGE and transferred to an Immobilon-P membrane (Millipore). The membrane was incubated with primary antibody at 4°C overnight and with secondary antibody at room temperature for 1 h. An ECL reagent (Millipore) was added for chemiluminescent detection. The primary antibodies used for immunoblotting were as follows: rabbit anti-USP17 (1:1000), anti-GAPDH (1:5000), anti-HA (1:3000), anti-SNAI1 (1:500), anti-N-cadherin (1:1000), anti-E-cadherin (1:1000), and anti-vimentin (1:1000).

### Statistical Analysis

Statistical analyses were performed with GraphPad 7 (GraphPad Software, San Diego, CA, USA). The 2^-ΔΔCT^ method was used to analyze the relative expression level of RNAs. All data were expressed as means ± SD of three or more independent experiments. Sample sizes were selected based on previous publications or related articles. A two-tailed Student’s *t*-test was used for comparison between two groups. Multiple comparisons were made using one-way ANOVA. Statistical significance was determined at *P* < 0.05.

## Results

### *LINC02487* Was Downregulated in OSCC Samples and Cell Lines, and LINC02487 Expression Levels Were Correlated With Cancer Metastasis

**Figure 1:**
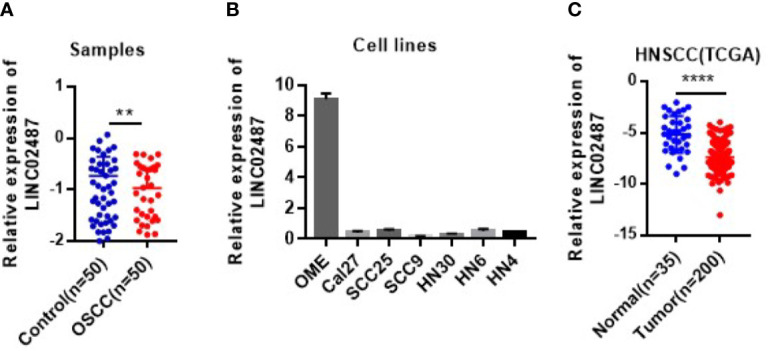
The expression levels of *LINC02487* in tissue samples and cell lines. **(A)** The expression of *LINC02487* was downregulated in OSCC samples compared with paired adjacent normal tissues. **(B)** The relative expression levels of *LINC02487* were significantly lower in the OSCC cell lines Cal27, SCC25, SCC9, HN30, HN6, and HN4 than in the immortalized normal human oral mucosal epithelial cell line hTERT-OME. **(C)** The expression of *LINC02487* was downregulated in HNSCC samples compared with normal control samples in TCGA data sets. HNSCC, head and neck squamous cell carcinoma; ns, not significant; **P < 0.01, ****P < 0.0001.

To determine the status of *LINC02487* in OSCCs, we used RT-qPCR to compare the relative expression levels of *LINC02487* in 50 paired OSCC cancer tissues and adjacent normal tissues. The characteristics of all the patients and the relative *LINC02487* expression levels in the different groups are summarized in **Table 1**. The results showed that *LINC02487* expression was significantly downregulated in OSCC samples compared with that in paired adjacent normal tissues (*P* < 0.01) (**Figure 1A**). The average fold change was 51.4. Moreover, the expression level of *LINC02487* differed significantly between patients with or without near and/or distant metastasis, indicating that the *LINC02487* level was potentially associated with cancer metastasis (**Table 1**). We also evaluated *LINC02487* expression in OSCC cell lines. The results demonstrated that *LINC02487* expression was also downregulated in the OSCC cell lines HN4, HN6, HN30, SCC9, SCC25, and Cal27 compared with that in immortalized normal oral epithelial cell line hTERT-OME (**Figure 1B**). To confirm these results, we also analyzed the relative expression level of *LINC02487* in TCGA data and found that it was markedly lower in HNSCC than in normal controls (*P* < 0.0001) (**Figure 1C**).

### *LINC02487* Was Localized to the Cytoplasm and Aggregated Around the Nuclear Membrane

**Figure 2:**
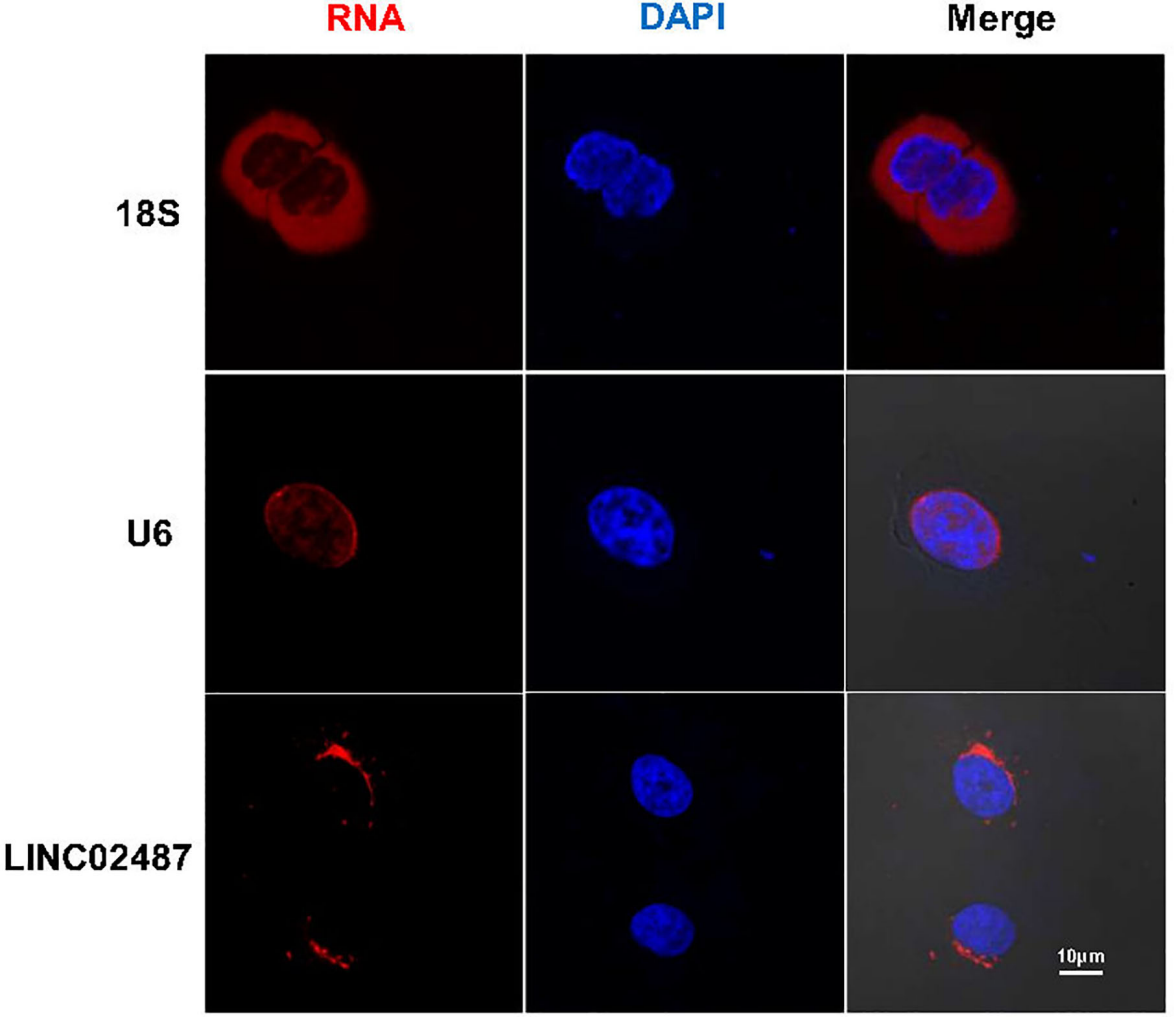
Long noncoding RNA *LINC02487* was mainly localized to the cytoplasm and aggregated around the nuclear membrane. 18S ribosomal RNA and *U6* were used as controls. ns, not significant.

We next performed FISH to explore the subcellular localization of *LINC02487.* Confocal microscopy analysis showed that the *LINC02487* signal was mainly localized to the cytoplasm and aggregated around the nuclear membrane (**Figure 2**).

### Overexpression of *LINC02487* Inhibited Cell Proliferation

**Figure 3:**
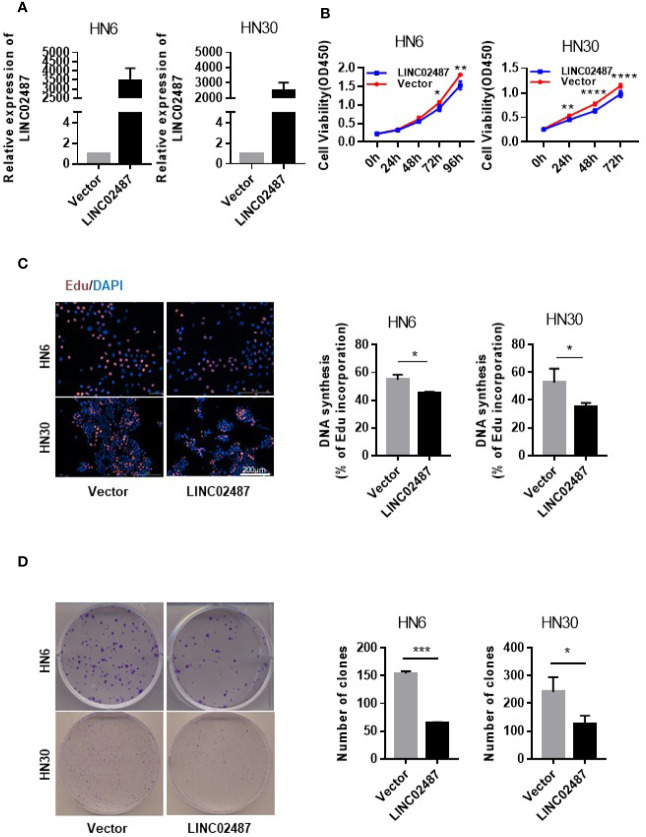
Overexpression of *LINC02487* inhibited cell proliferation in the OSCC cell lines HN6 and HN30. **(A)** The relative expression level of *LINC02487* after lentiviral plasmid transfection. **(B, C)** CCK-8 and EdU staining assays showed that cell proliferation was inhibited after *LINC02487* overexpression. **(D)** Overexpression of *LINC02487* also diminished the colony-forming ability of OSCC cells. ns, not significant; *P < 0.05, **P < 0.01, ***P < 0.001, ****P < 0.0001.

To investigate the function of *LINC02487* in OSCCs, we established *LINC02487*-overexpressing HN6 and HN30 OSCC cell lines through the lentiviral transfection of pCMV-puro plasmids. RT-qPCR results showed a more than 3000-fold stable overexpression of *LINC02487* after transfection in HN6 cells and a more than 2000-fold overexpression in HN30 cells (**Figure 3A**). Next, we compared the phenotypic changes occurring in *LINC02487*-overexpressing cells compared with control cells. CCK-8 and EdU staining assays both revealed that cell viability was decreased in *LINC02487*-overexpressing cells (**Figures 3B, C**). Cell apoptosis was also examined through analysis of both caspase-3 activity and Annexin V/FITC staining in the two cell lines. Overall, no significant differences in the rate of apoptosis were found between the two cell lines (**Supplementary Figure 1A, B**). A colony formation assay showed that fewer clones formed with *LINC02487* overexpression (**Figure 3D**). Moreover, flow cytometry analysis demonstrated that the ratio of cells in the G1 phase of the cell cycle was increased in *LINC02487*-overexpressing cells (**Supplementary Figure 1C**). In summary, overexpression of *LINC02487* suppressed cell proliferation in OSCC cell lines.

### Overexpression of *LINC02487* Significantly Inhibited Cell Migration and Invasion in OSCC Cells

**Figure 4:**
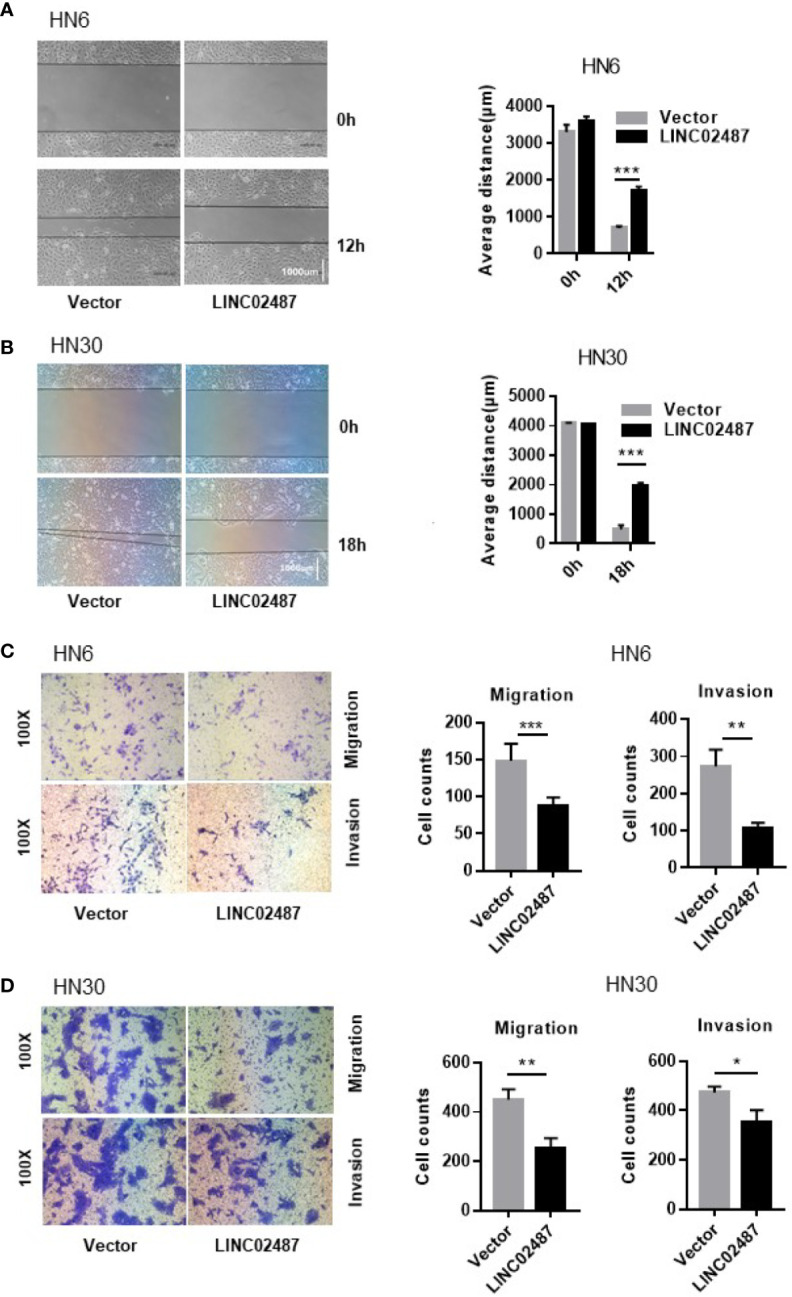
Overexpression of *LINC02487* inhibited the migratory and invasive abilities of the OSCC cell lines HN6 and HN30. **(A, B)** A wound healing assay showed that overexpression of *LINC02487* affected cell migration. **(C, D)** A transwell assay showed that both cell migration (without Matrigel) and invasion (with Matrigel) were inhibited after *LINC02487* overexpression. ns, not significant; *P < 0.05, **P < 0.01, ***P < 0.001.

To further explore the function of *LINC02487*, we investigated the effect of overexpressing *LINC02487* on the migratory and invasive capabilities of HN6 and HN30 cells. We first analyzed cell migration using a wound healing assay. A significant increase in the average migrated distance was observed in *LINC02487*-overexpressing cells compared with that in mock control cells (**Figures 4A, B**). Transwell assays with or without Matrigel-coated upper chambers showed that both migratory and invasive abilities were significantly suppressed in *LINC02487*-overexpressing cells (**Figures 4C, D**).

### *LINC02487* Directly Bound to USP17

**Figure 5:**
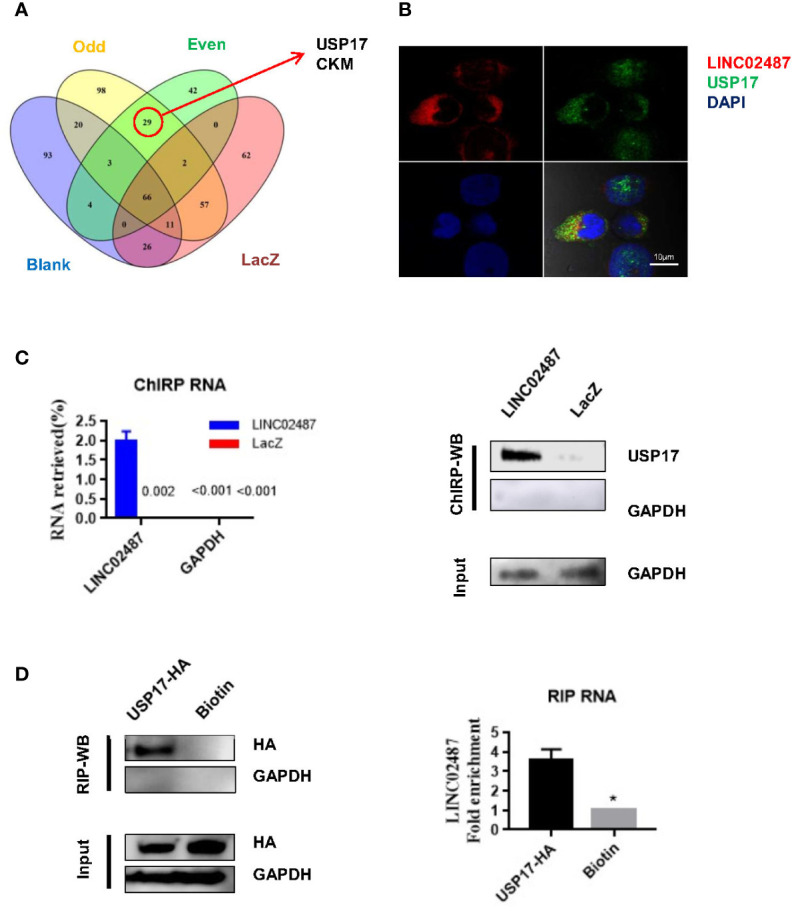
*LINC02487* directly bound USP17. **(A)** Chromatin isolation by RNA purification–mass spectrometry (ChIRP–MS) results show that *LINC02487* binds primarily to two proteins: USP17 and CKM. **(B)** Colocalization of *LINC02487* and USP17 using confocal microscopy. **(C)** ChIRP-western blot demonstrated the direct binding of *LINC02487* and USP17; LacZ was used as control. **(D)** RNA immunoprecipitation confirmed the binding of *LINC02487* and USP17. Biotin beads were used as control. ns, not significant; *P < 0.05.

To explore the mechanisms underlying *LINC02487* function, we purified its binding complexes and identified *LINC02487*-binding proteins by mass spectrometry (ChIRP–MS). As previously reported, peptides detected in both the even and odd groups but not in the LacZ and blank groups were considered to be *LINC02487*-binding proteins. Twenty-nine peptides representing two protein families were detected with most (26 out of 29) belonging to the ubiquitin carboxyl-terminal hydrolase 17 (USP17) family (**Figure 5A**). The mass spectrometry data is shown in the **Supplementary data**. Analysis of double-fluorescence staining by confocal microscopy showed that *LINC02487* and USP17 displayed a high ratio of overlap in the cytoplasm (**Figure 5B**). This result can also be confirmed by western blotting (ChIRP–WB) assay (**Figure 5C**). To verify this result, we then performed RNA immunoprecipitation using HA-tagged USP17; the complex was purified using HA magnetic beads, followed by detection of *LINC02487* by RT-qPCR. The result shows that *LINC02487* was more than three times higher in the USP17-HA group (**Figure 5D**). Together, these results demonstrate that *LINC02487* bound directly to USP17 and suggests that *LINC02487* may modulate cell functions through USP17.

### *LINC02487* Suppressed Tumor Migration and Invasion Through EMT-Related Processes

**Figure 6:**
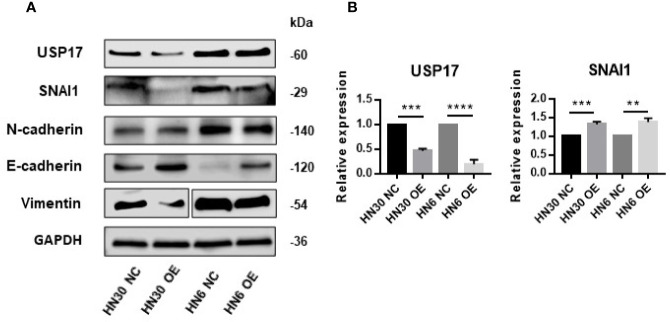
Overexpression of *LINC02487* suppressed EMT through the USP17–SNAI1 axis. **(A)** The protein expression of USP17 and EMT-related markers in the OSCC cell lines HN30 and HN6. Blots for vimentin were spliced from two scans due to expression differences between the two cell lines. **(B)** The expression levels of *USP17* and *SNAI1* mRNA in the cell lines HN30 and HN6. ns, not significant; **P < 0.01, ***P < 0.001, ****P < 0.0001.

USP17 is reported to regulate cancer metastasis through modulation of EMT (38, 39). To determine whether *LINC02487* modulates OSCC cell function through this path, we examined EMT-related markers in *LINC02487*-overexpressing cell lines HN6 and HN30. The level of the epithelial marker E-cadherin was increased compared with that in control cells, and the levels of the mesenchymal markers N-cadherin and vimentin were decreased after *LINC02487* overexpression (**Figure 6A**). We also find that the expression of the key EMT regulator SNAI1 was increased at the mRNA level but decreased at the protein level; meanwhile, the expression of USP17 was decreased at both the mRNA and protein level (**Figures 6A, B**). This result could be explained by the posttranslational regulation resulting from the activity of the deubiquitinating enzyme USP17.

### *LINC02487* Inhibited EMT Through the USP17–SNAI1 Axis

**Figure 7:**
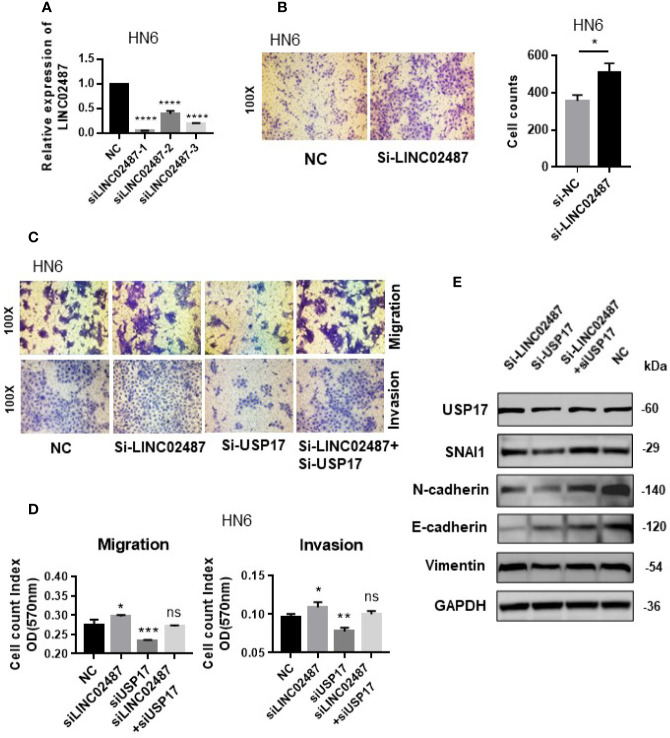
**(A)** The efficiency of the three *LINC02487* siRNAs in the OSCC cell line HN6; Si-LINC02487-1 was chosen for subsequent experiments. **(B)** The knockdown of *LINC02487* increased cell migration. **(C, D)** The knockdown of *LINC02487* increased the migratory and invasive capacity of the OSCC cell line HN6 although knockdown of USP17 suppressed these processes. Simultaneous knockdown of *LINC02487* and USP17 restored cell functions. **(E)** Western blot assay showed that the changes in cell migratory ability were mediated through EMT. ns, not significant; *P < 0.05, **P < 0.01, ***P < 0.001, ****P < 0.0001.

We next determined whether the effect of *LINC02487* on cell mobility was mediated through the USP17–SNAI1 axis using the OSCC cell line HN6. First, we assessed the silencing efficiency of three siRNAs and selected siRNA-1 for subsequent experiments (**Figure 7A**). A transwell assay showed that knockdown of *LINC02487* increased the migratory ability of the HN6 cells (**Figure 7B**). We then performed a series of transwell and western blot assays to unravel the associated regulatory network. The results showed that knocking down USP17 decreased cell migration and invasion, and this effect could be rescued by simultaneously knocking down *LINC02487* and USP17 (**Figures 7C, D**). Western blotting assay further revealed that this rescue was mediated through the EMT process. When USP17 was knocked down, cells presented a more epithelial-like phenotype, and this effect could be rescued by silencing *LINC02487* (**Figure 7E**).

## Discussion

LncRNA expression contributes significantly to the whole transcriptome and is dysregulated in many cancer types, including OSCC (15, 40, 41). LncRNAs can be epigenetic, transcriptional, and posttranscriptional regulators in various cancer types (42–45). Studies have reported that *LINC02487* (previously known as *LOC441178*) is significantly downregulated in OSCC samples when compared with samples obtained from healthy controls (18, 19), and *LINC02487* expression levels were found to be associated with postoperative survival time, indicating that it has potential for use as a biomarker for OSCC diagnosis and prognosis. In this study, we find that *LINC02487* expression was significantly decreased in OSCC samples derived from a Chinese population; this is consistent with previous studies and confirms the similarity in *LINC02487* OSCC expression pattern between different continents and populations. As lncRNAs have been reported to display additional tissue-specific expression patterns (10, 40), using TCGA data, we explored the *LINC02487* status in different cancer types through TANRIC, an open-access resource for interactive exploration of lncRNAs. The analysis indicates that *LINC02487* was also downregulated in HNSCC, but upregulated in breast cancer and kidney renal papillary cell carcinoma when compared with nontumor controls (data not shown). This result is also consistent with previous reports and is further confirmed in OSCC cell lines in a comparative analysis with immortalized normal oral epithelial cells. Additionally, the expression level of *LINC02487* is associated with cancer metastasis, indicating that this lncRNA may be involved in OSCC development.

Functionally, overexpression analysis shows that the increased expression of *LINC02487* influences several cell characteristics, including viability, mobility, and cell cycle progression. In particular, *LINC02487* gain-of-function inhibits cell proliferation, migration, and invasion, indicating that this lncRNA might play a role as a tumor suppressor in OSCC.

The functional mechanisms of lncRNAs are determined by their cellular localization, at least partially (24, 46). Most lncRNAs are predominantly localized to the chromatin and nucleus and have lower expression levels than coding mRNAs (10). In this study, we use FISH to show that *LINC02487* was mostly localized to the cytoplasm, and aggregated around the nuclear membrane. This suggests that *LINC02487* acts mainly at the posttranscriptional or posttranslational level.

We performed ChIRP–MS to explore the mechanisms underlying the cell functions of *LINC02487* and find that *LINC02487* bound directly to USP17. According to UniProt, USP17 is mainly found in the nucleus and endoplasmic reticulum (34, 47). In our study, we demonstrate that *LINC02487* was located in the cytoplasm, aggregated near the nuclear membrane, where USP17 is reported to also be localized (47). The binding of *LINC02487* and USP17 was further verified by ChIRP–WB and RIP. These results indicate that *LINC02487*-regulated cell functions may be mediated through USP17.

USP17 belongs to a subfamily of deubiquitinating enzymes (DUBs). These enzymes are key regulators of posttranslational protein modifications through ubiquitination in many cellular processes, including transcription, DNA repair, cell-cycle progression, and apoptosis (48, 49). Additionally, as a cytokine-inducible DUB, USP17 is also reported to inhibit proliferation, increase apoptosis, and regulate the G1/S phase transition of the cell cycle (50–52). Several studies also indicate that USP17 may be involved in cancer metastasis through EMT regulation (39, 49, 53). Tumor cells undergoing EMT acquire a mesenchymal phenotype and begin to express markers such as N-cadherin and lose the expression of epithelial markers such as E-cadherin, leading to tumor progression, invasion, and eventually distant metastasis. EMT is an essential step in cancer metastasis. Emerging evidence has shown that lncRNAs influence the EMT process mainly through cross-talk with master regulators, such as SNAI1 and ZEB (21, 25, 30, 54, 55). In this study, western blot analysis showed that overexpressing *LINC02487* led to the upregulation of the epithelial marker E-cadherin and downregulation of the mesenchymal markers N-cadherin and vimentin, indicating that *LINC02487* regulates cell invasion and migration through the EMT process. Mechanistically, we also tested the expression of USP17 and SNAI1 in the OSCC cell lines HN6 and HN30. Overexpression of *LINC02487* suppressed the expression of USP17 at both the mRNA and protein level. Interestingly, however, we found that overexpressing *LINC02487* led to a reduction in the protein level, but not the mRNA level, of SNAI1. The SNAI1 protein is highly unstable and can be posttranslationally degraded through the ubiquitin–proteasome pathway. USP17 is reported to exert its role in EMT through the posttranslational deubiquitination and stabilization of SNAI1. Inhibition of USP17 promotes SNAI1 degradation, thereby suppressing breast cancer invasion and metastasis (49, 53). Our findings are consistent with previous reports.

We further tested the cell function of *LINC02487* in the OSCC cell line HN6. As USP17 is known to have several cellular functions, we only tested its role in cell mobility. First, a transwell assay revealed that silencing *LINC02487* led to an increase in cell migration, whereas knocking down USP17 suppressed cell migration and invasion, and this effect could be rescued by the loss of *LINC02487*. We then examined the expression levels of EMT-related markers with the results demonstrating that the expression levels of these proteins were changed accordingly. When we silenced *LINC02487*, OSCC cells showed increased mesenchymal characteristics, and when USP17 was silenced, OSCC cells displayed a more epithelial phenotype. However, this effect could also be rescued by the loss of *LINC02487*. Together, these results indicate that *LINC02487* may be a negative regulator of USP17, and has a role in the EMT process through the USP17–SNAI1 axis. McFarlane et al. identify that USP17 is highly expressed in several tumor biopsies, and USP17 depletion significantly impairs G1-S phase transition and blocks cell proliferation (56). In our study, we observed that the overexpression of *LINC02487* exerted similar effects on cells (**Supplementary Figure 1C**). Whether it was also through the *LINC02487*-USP17 axis has yet to be proved. Altogether, we demonstrated that *LINC02487* binds directly to, and negatively regulates, USP17 in OSCC cells.

In conclusion, our study confirms that long noncoding RNA *LINC02487* is downregulated in OSCC samples and cell lines, and its expression level is correlated with cancer metastasis. We further show that *LINC02487* binds directly to USP17 and acts as a tumor suppressor through the USP17–SNAI1 axis.

## Data Availability Statement

The raw data supporting the conclusions of this article will be made available by the authors, without undue reservation.

## Ethics Statement

The studies involving human participants were reviewed and approved by Ethics Committee of Zhengzhou University. The patients/participants provided their written informed consent to participate in this study.

## Author Contributions

Conception and design: LF, WC, and WQ. Development of methodology: LF and JZ. Data acquisition: LF, MS, and FQ. Data analysis and interpretation: LF, JZ, and WC. Writing, reviewing, and/or revision of the manuscript: LF, WC, and JZ. Study supervision: WQ and WC. All authors contributed to the article and approved the submitted version.

## Funding

This paper was funded by grants from the Oral and Maxillofacial Surgery Academician Workstation of Zhengzhou (152PYSGZ040), the National Natural Science Foundation of China (81874126), and Shanghai Municipal Science and Technology Commission Funded Projects (18DZ2291500, 16140903300).

## Conflict of Interest

The authors declare that the research was conducted in the absence of any commercial or financial relationships that could be construed as a potential conflict of interest.
