# Supplementary figures and images for: Tumor Suppressor LINC02487 Inhibits Oral Squamous Cell Carcinoma Cell Migration and Invasion Through the USP17–SNAI1 Axis
Source: Front Oncol. 2020 Oct 29;10:559808. doi: 10.3389/fonc.2020.559808 (PMC7658685; doi:10.3389/fonc.2020.559808)

RT: 0.00 - 60.00

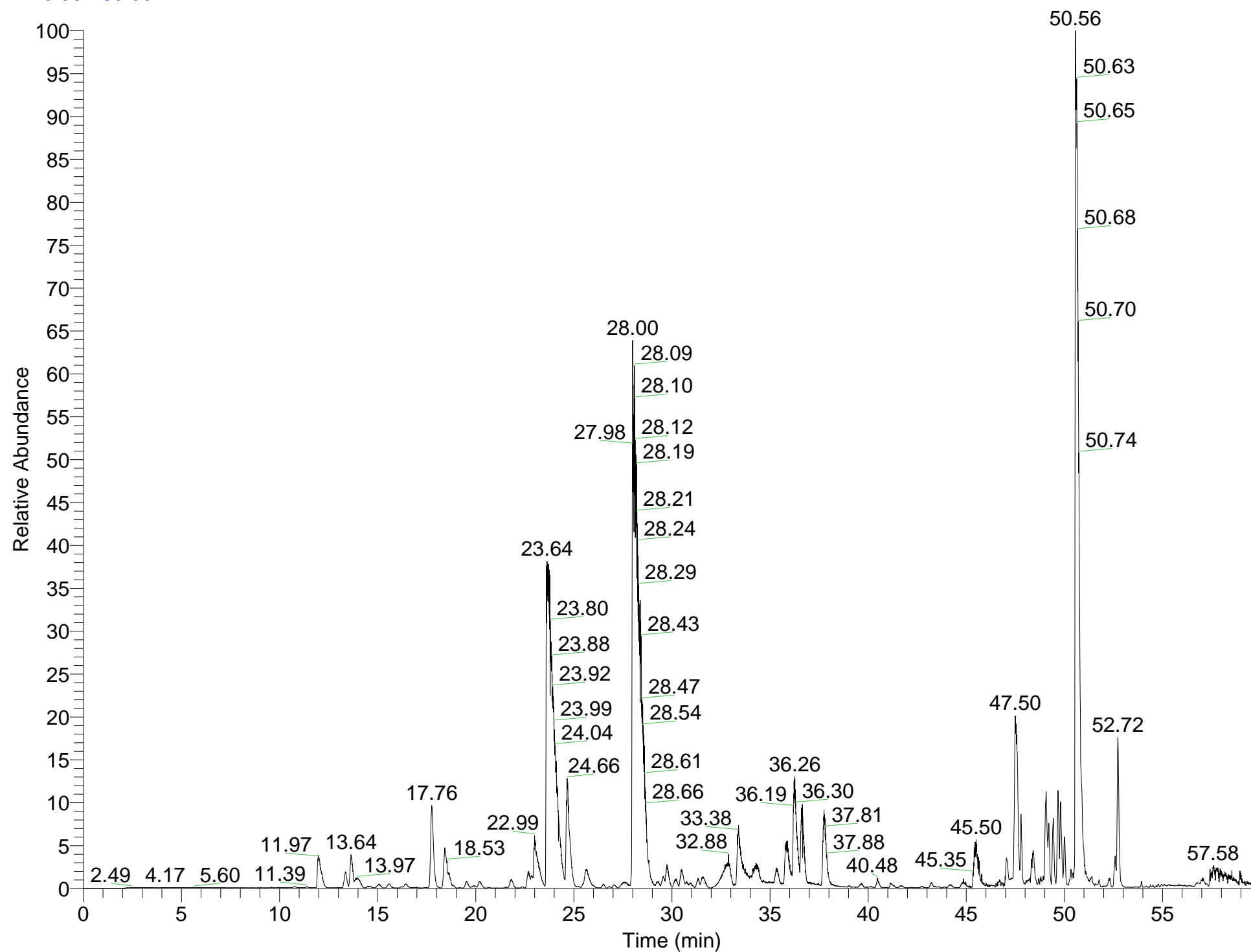

NL: 1.64E9

Base Peak F: FTMS

+ p NSI Full ms

[300.0000-

1800.0000] MS

R20180901112\_BLA

NK

Supplement: Supplementary file 1 [file DataSheet_1.zip › Mass spectrometry/base peak/R20180901112_BLANK.pdf]

RT: 0.00 - 60.00

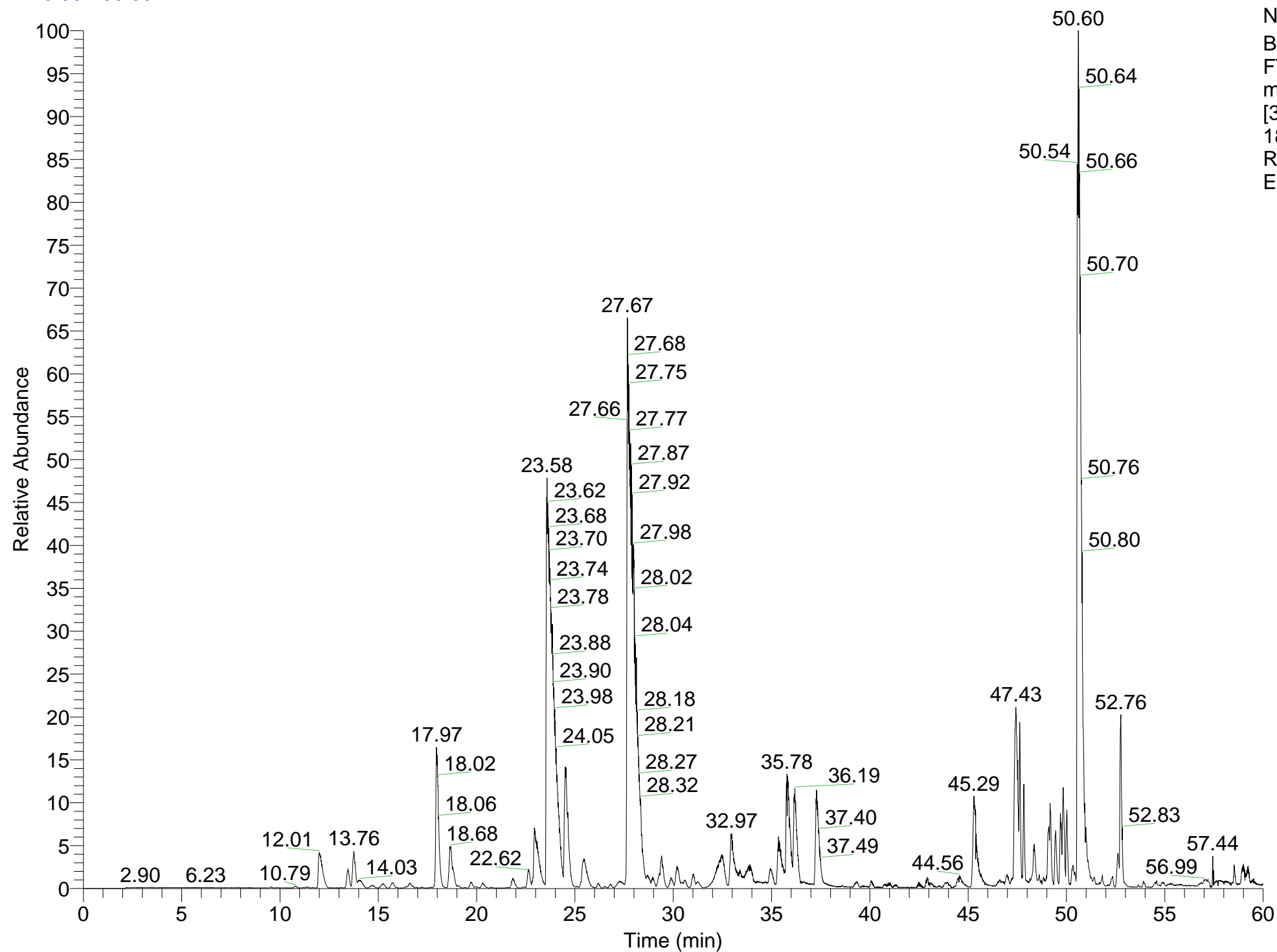

NL: 1.60E9

Base Peak F:

FTMS + p NSI Full  
ms[300.0000-  
1800.0000] MSR20180901112\_EV  
EN

Supplement: Supplementary file 1 [file DataSheet_1.zip › Mass spectrometry/base peak/R20180901112_EVEN.pdf]

RT: 0.00 - 60.00

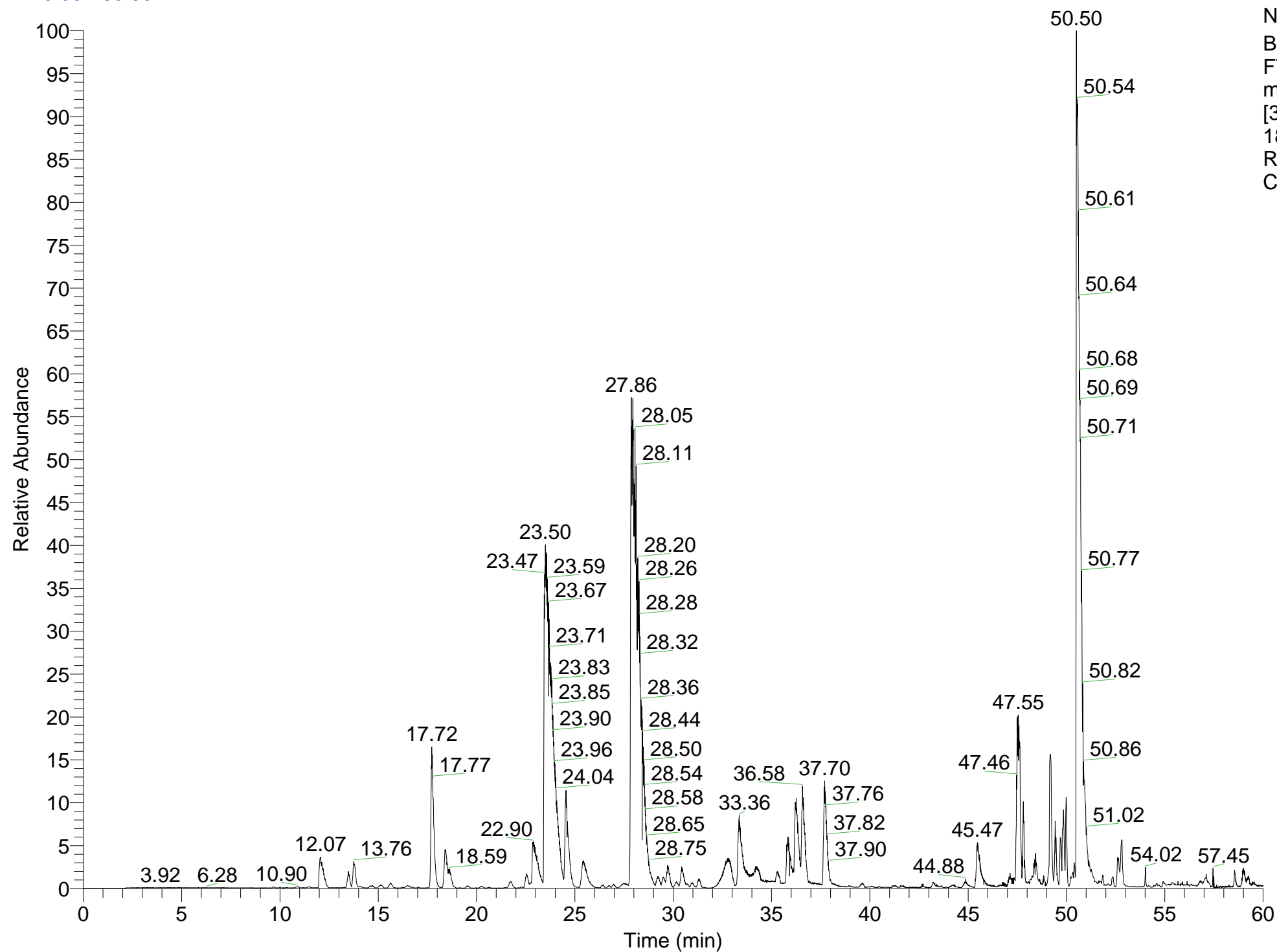

NL: 1.87E9

Base Peak F:

FTMS + p NSI Full  
ms[300.0000-  
1800.0000] MSR20180901112\_LA  
CZ

Supplement: Supplementary file 1 [file DataSheet_1.zip › Mass spectrometry/base peak/R20180901112_LACZ.pdf]

RT: 0.00 - 60.00

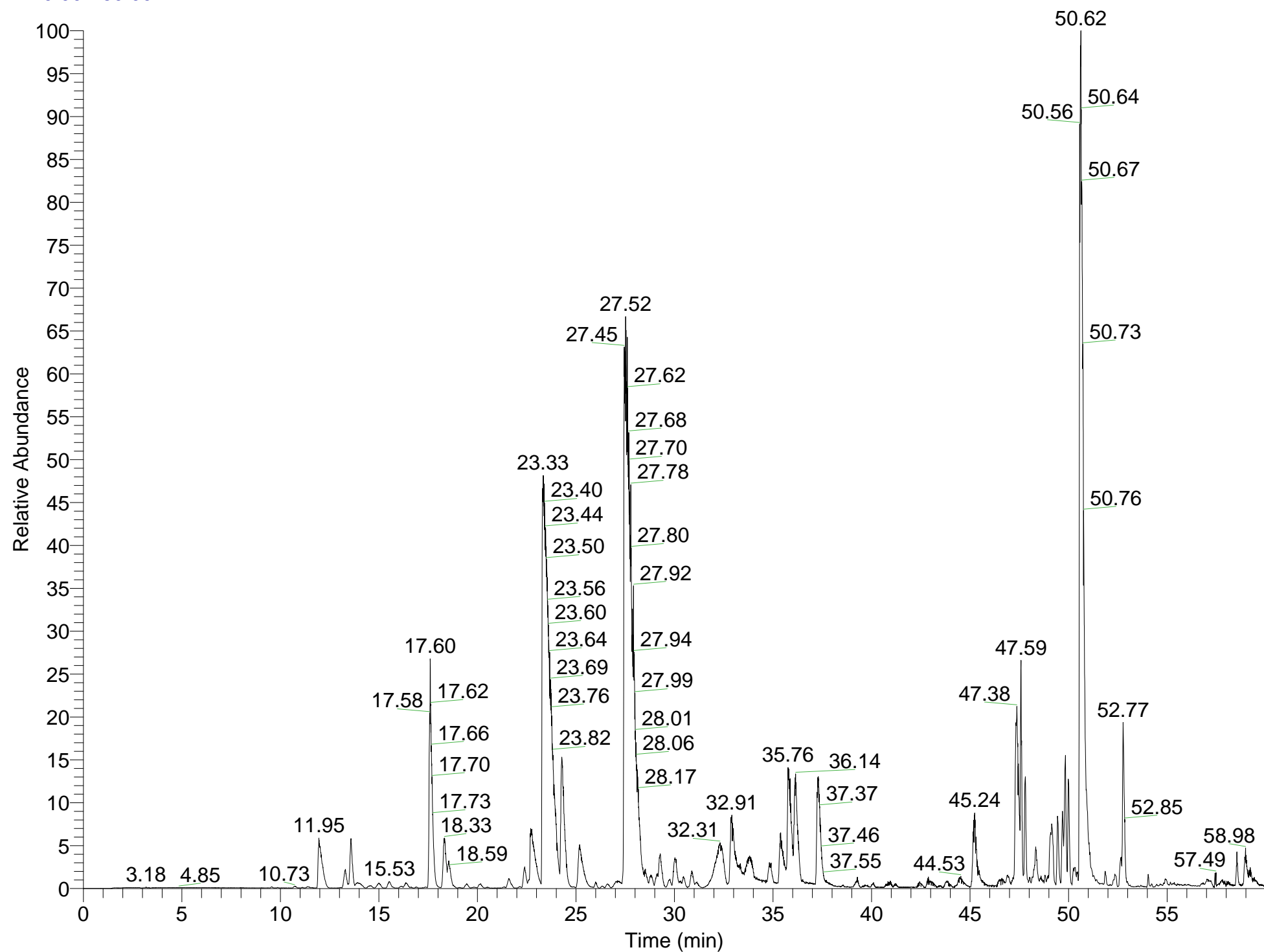

NL: 1.67E9

Base Peak F:

FTMS + p NSI Full

ms

[300.0000-  
1800.0000] MSR20180901112\_OD  
D

Supplement: Supplementary file 1 [file DataSheet_1.zip › Mass spectrometry/base peak/R20180901112_ODD.pdf]

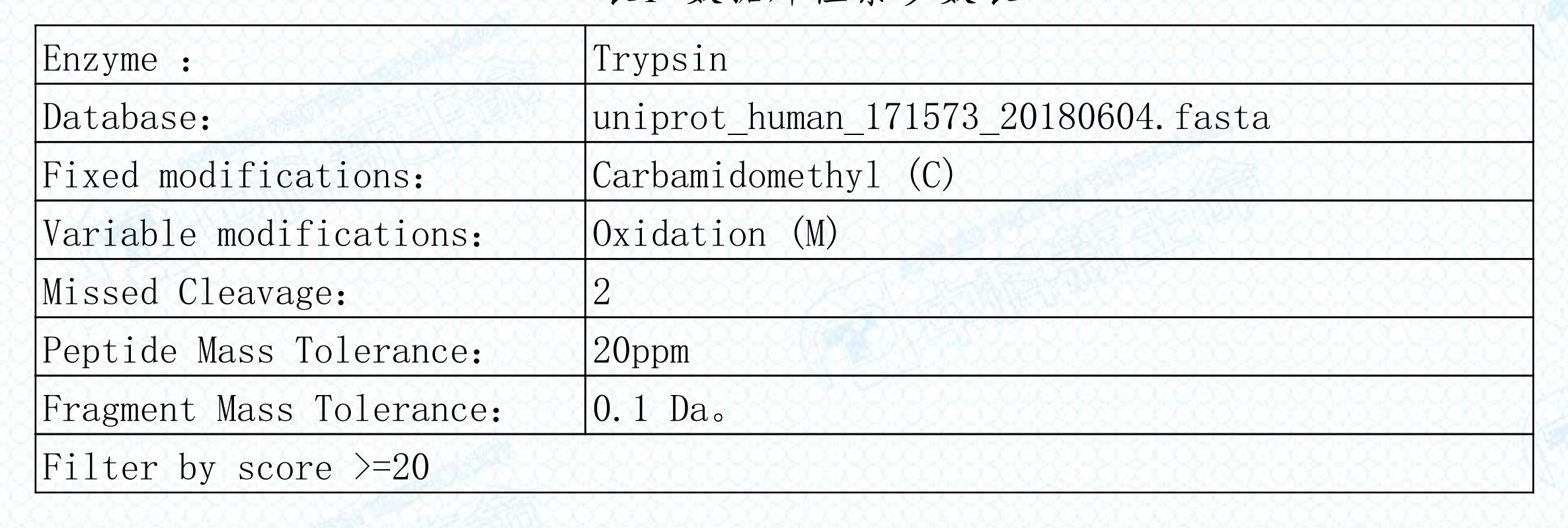

Supplement: Supplementary file 1 [file DataSheet_1.zip › Mass spectrometry/Parameters for searching.jpg]

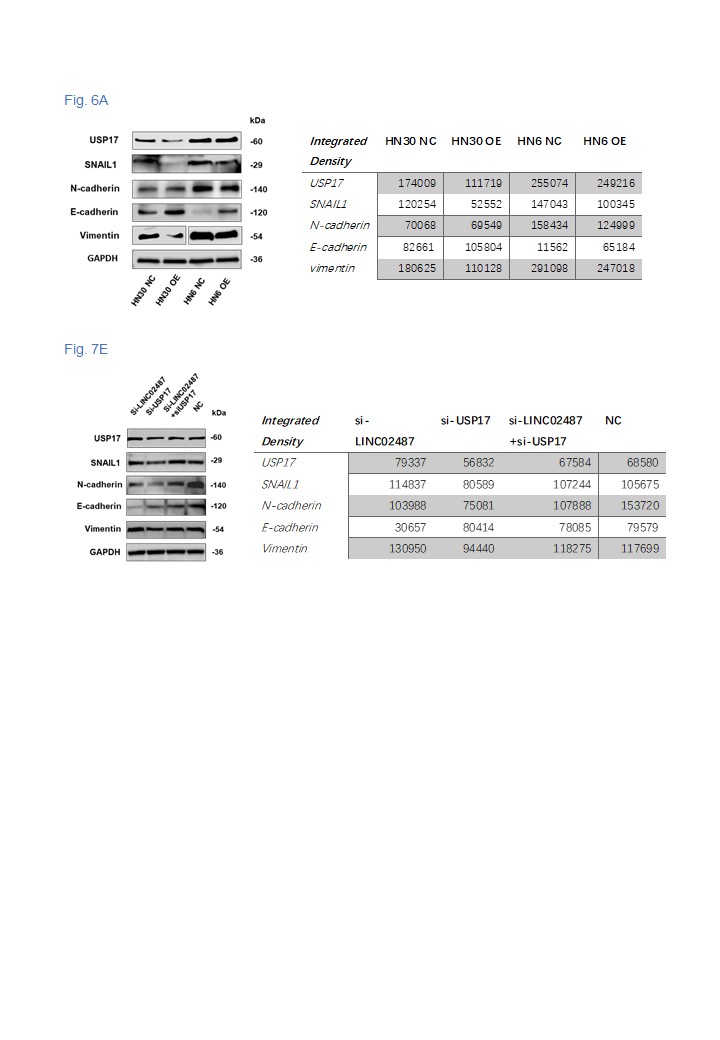

Supplement: Supplementary file 2 [file Image_1.jpeg]

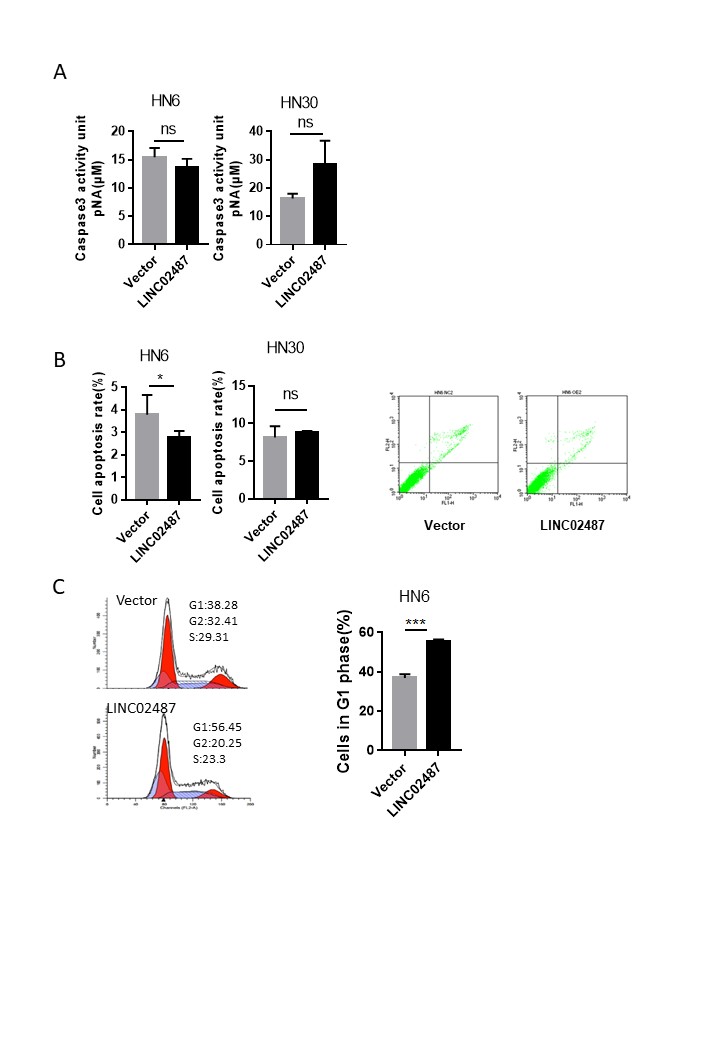

Supplement: Supplementary file 3 [file Image_2.jpeg]
