# Supplementary material for: Tumor Suppressor LINC02487 Inhibits Oral Squamous Cell Carcinoma Cell Migration and Invasion Through the USP17–SNAI1 Axis
Source: Front Oncol. 2020 Oct 29;10:559808. doi: 10.3389/fonc.2020.559808 (PMC7658685; doi:10.3389/fonc.2020.559808)
Supplement: Supplementary file 4 [file Table_1.docx]

Supplementary Material

# Supplementary Figures

**Supplementary Figure 1.** **(A)** An activated caspase-3 detection assay showed that cell apoptosis was no significance after overexpression of *LINC02487* in the OSCC cell lines HN6 and HN30. **(B)** Flow cytometric analysis of Annexin V/FITC staining also showed that cell apoptosis was slightly inhibited in HN6 cells but no significance in HN30 cells. **(C)** Cell-cycle analysis showed an increase in the number of cells in the G1 phase in *LINC02487*-overexpressing HN6 cells. ns: not significant; **P* < 0.05, ***P* < 0.01, ****P* < 0.001, *****P* < 0.0001.

**Supplementary Figure 2.** Quantitative analyses of the western blotting results presented in **Figures 6A** and **7E**.

**Supplementary file: Mass spectrometry**. The primary results of ChIRP-MS after searching the UniProt database.
